# Teaching Aspects of Antibiotics and Antimicrobials to the Food Science Student through a Combination Wet Lab and *In Silico* Activity

**DOI:** 10.1128/jmbe.00157-21

**Published:** 2021-06-30

**Authors:** Tahl Zimmerman, Mariama Ibrahim, Salam A. Ibrahim

**Affiliations:** a Food Microbiology and Biotechnology Laboratory, Department of Family and Consumer Sciences, College of Agriculture and Environmental Sciences, North Carolina A&T State University, Greensboro, North Carolina, USA

## Abstract

To prevent the growth of food pathogens and food spoilage organisms, antimicrobials are used to disinfect surfaces or are added to food. In light of the important role that antibiotics and antimicrobials play in food safety, food science students need a deep understanding of how these chemicals function. We describe here a combined wet lab and *in silico* laboratory experience designed to help students visualize two biochemical concepts explaining antibiotic activity: (i) bacteriolytic versus bacteriostatic activity and (ii) competitive versus noncompetitive inhibition. This laboratory experience was implemented for students enrolled in the Introduction to Food Science course in Family and Consumer Sciences at North Carolina A&T State University.

## INTRODUCTION

Antibiotics are chemicals produced by microorganisms that kill other microorganisms (1). A hallmark of modern medicine is the use of antibiotics to fight infections, leaving the host unharmed (2). Antimicrobials function similarly but refer to any synthetic, semisynthetic, or natural substance (3). To prevent the growth of food pathogens and food spoilage organisms, antimicrobials are used to disinfect surfaces or are added to food. The mechanism of action of an antimicrobial added to food is a food safety issue, because with some food pathogens (e.g., Escherichia coli), the presence of even a single live cell is intolerable, while others can be tolerated at certain levels without causing illness (e.g., Salmonella). Therefore, how an antimicrobial interacts with a tolerable or intolerable pathogen will determine how it should be employed. We describe here a laboratory experience designed to help students visualize two biochemical concepts explaining antibiotic/antimicrobial activity: (i) bacteriolytic versus bacteriostatic activity and (ii) competitive versus noncompetitive inhibition. This laboratory experience was implemented for students enrolled in the Food Safety and Sanitation course at North Carolina A&T State University.

## BACTERIOLYTIC VERSUS BACTERIOSTATIC AGENTS

Antimicrobial agents can be bacteriostatic or bacteriolytic. Bacteriostatic agents inhibit the growth and reproduction of bacteria without killing them and, therefore, would be used in cases of pathogens that cannot be allowed to grow beyond a certain cutoff value. Bacteriostatic agents function by blocking protein production and DNA replication or cellular metabolism. Bacteriolytic agents, such as penicillin, kill cells outright by inducing digestion of the cell wall, leading to lysis. These agents would be used in cases where a single live pathogenic cell cannot be tolerated. Lysis of bacterial cells can be monitored by visible-range spectrophotometry, which can measure the turbidity of a solution. The level of turbidity of a bacterial culture is directly proportional to the number of intact cells. A drop in turbidity indicates lysis. Meanwhile, a constant turbidity is an indicator of a bacteriostatic mechanism. Penicillin functions by binding to proteins responsible for blocking the cross-linking of the peptidoglycan molecules in a cell wall, weakening the cell wall and leaving the cell to succumb to osmotic pressure that causes the cell to rupture.

## NONCOMPETITIVE VERSUS COMPETITIVE INHIBITORS

Many antibiotic and antimicrobial agents are enzyme inhibitors that function by inhibiting a key enzyme involved in cell metabolism. An enzyme is a protein molecule that catalyzes a chemical reaction and has one or more substrates. A substrate is the chemical that is modified by the enzyme. Every enzyme has an active site, which is a cavity in the structure that holds the substrates in an orientation that allows the chemical reaction to proceed. One example of an enzyme inhibitor is the choline kinase inhibitor (CKI), which blocks the activity of choline kinase (4, 5). Choline kinase catalyzes the production of phosphocholine via a phosphotransfer from ATP to choline. Phosphocholine is, in turn, a building block of lipoteichoic acid, an important component of the bacterial cell wall (5). The activity of this enzyme is critical for the successful growth and division of a bacterial cell (4, 5).

Two major types of enzyme inhibitors are noncompetitive and competitive inhibitors. Noncompetitive inhibitors function by binding a site on the enzyme outside the active site, which prevents the active site from functioning. Competitive inhibitors function by entering the active site of an enzyme and displacing the substrate. Docking software, such as Patchdock, can be used to assess the possibility that an inhibitor is competitive (6) by determining how well the molecule displaces the substrate.

## ACTIVITY

Students came into the class having watched from home a set of YouTube videos that went over a demonstration of the wet lab activity and basic lab safety rules (see Appendix S4 in the supplemental material). Biosafety level 2 (BSL-2) safety rules were also reviewed in class (Appendix S7), followed by a lecture on antibiotics and their classifications.

### Cell culture activity

To gain an understanding of the key differences between bacteriolytic and bacteriostatic antibiotic activity, students tested the effects of penicillin, CKI, and the two combined on the nonpathogenic R6 strain of Streptococcus pneumoniae (Appendix S2). Based on the information provided to them about penicillin and CKI, the students were first prompted to hypothesize what the effects of each condition would be. Penicillin is a known bacteriolytic, while the mechanism of CKI was left unexplained. Students observed the optical densities of cultures treated with CKI or penicillin densities using a spectrophotometer (Appendix S2). Students were asked to analyze the data as part of a laboratory report and to come to a conclusion about how to classify penicillin and CKI (bacteriolytic versus bacteriostatic; Appendix S3).

### Molecular modeling activity

To gain an understanding of the key differences between competitive and noncompetitive inhibitors, students modeled CKI binding to the active site of the choline kinase enzyme of S. pneumoniae using the *in silico* tools PyMOL and Patchdock to determine if CKI could fit into the active site of choline kinase. The structure files in this activity (docking.zip) were generated by the instructors in PyMOL using the structural information found on the RCSB website (https://www.rcsb.org/structure/4R77).

This activity consisted of six parts: (i) inspection of the structure of choline kinase with choline; (ii) definition of the choline kinase active site; (iii) inspection of CKI; (iv) docking of CKI to the active site of choline kinase; (v) inspection of structure of choline kinase in complex with CKI; and (vi) comparison with the structure of choline kinase in complex with its natural substrate, choline (Appendix S2 and S7).

## ASSESSMENT

Students took a pre/postexam administered using the online service Kahoot.it. The experience took on game-show style, as students saw the percent breakdown of student answers in real time (see Appendix S1 for exam questions and Appendix S4 for a link to the Kahoot.it exam). Lab reports were also assessed (Appendix S3), as was student performance (Appendix S8 and S10).

## HEALTH AND SAFETY

In the culturing activity, the nonvirulent R6 strain of S. pneumoniae was used. Nevertheless, this organism is BSL-2 (https://www.atcc.org/Products/All/BAA-255.aspx). To increase safety, the activity could be modified by preadding the antibiotics to the cultures, leaving the student with a sealed test tube with which to measure optical densities. A BSL-1 organism also could be substituted. The *in silico* exercise can be carried out at home using the online tools.

## CONCLUSIONS

This exercise teaches about the nature of bacteria and the link between controlling their growth and food safety. The instructions reinforced in the instructional videos, in-person lecture, and student activity guide were easily followed, and all participants successfully completed the activity.
