# Supplementary material for: Teaching Aspects of Antibiotics and Antimicrobials to the Food Science Student through a Combination Wet Lab and In Silico Activity
Source: J Microbiol Biol Educ. 2021 Jun 30;22(2):e00157-21. doi: 10.1128/jmbe.00157-21 (PMC8441997; doi:10.1128/jmbe.00157-21)
Supplement: Appendix 1, Appendix 2, Appendix 3, Appendix 4, Appendix 5, Appendix 6, Appendix 7, Appendix 8, Appendix 9, Appendix 10 — Appendix S1 Pre-experience exam. Appendix S2 Student guide handout. Appendix S3 Lab report rubric. Appendix S4 Important links. Appendix S5 Materials and solution recipes. Appendix S6 Laboratory report outline. Appendix S7 Information about docking activity. Appendix S8 BSL-2 norms. Appendix S9 Student performance rubric. Appendix S10 Results of assessments. Download JMBE00157-21_Supp_1_seq2.docx, DOCX file, 0.9 MB [file jmbe00157-21_supp_1_seq2.docx]

Appendices

Appendix 1: Pre-experience exam

Appendix 2: Student Guide Handout

Appendix 3: Lab Report Rubric

Appendix 4: Important Links

Appendix 5: Materials and solution recipes

Appendix 6: Laboratory report outline

Appendix 7: Information about Docking Activity

Appendix 8: BSL-2 norms.

Appendix 9: Student Performance Rubric

Appendix 10: Student Assessments

Docking Files: Docking.zip

**Appendix 1: Pre-experience exam (correct answer shown with a *)**

Can be found on the following link:

https://create.kahoot.it/v2/share/cf550be2-8791-4917-be3e-f0820e3b45f3


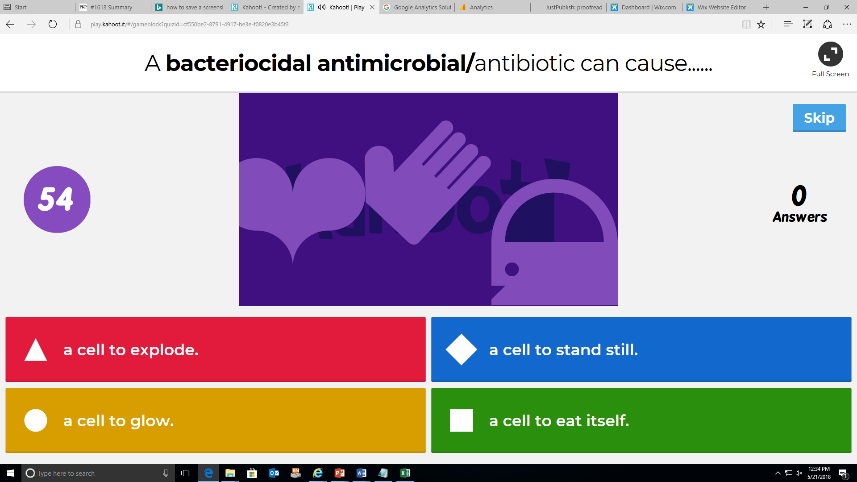


1. An antimicrobial/antibiotic can......

a) kill tumor cells.

b) kill viruses and bacteria.

c) kill fungi and bacteria. *

d) kill tumor cells and bacteria.

2. A bacteriostatic antimicrobial/antibiotic causes

a) the bacterial cell to break open

b) the bacterial cell to stop growing. *

c) the bacteria cell to mutate.

d) the bacterial to grow and divide.

3. A bacteriostatic antimicrobial/antibiotic......

a) Slows the metabolism of a bacterial cell. *

b) Causes bacterial cells to stick together.

c) Disrupts the cell wall of a bacterial cell.

d) Causes bacterial cells to stop moving.

4. A bacteriolytic antimicrobial/antibiotic can cause......

a) a cell to explode. *

b) a cell to stand still.

c) a cell to glow.

d) a cell to eat itself.

5. A bacteriolytic antibiotic/antimicrobial

a) Slows down the bacterial cell metabolism.

b) Causes a breakdown in the cell wall. *

c) Slows down the immune system.

d) Causes the cell to grow and divide.

6. Which kind of antimicrobial/antibiotic relies more on the immune system to have an effect?

a) bacteriostatic *

b) both

c) bacteriolytic

d) neither

7. Penicillins and its derivatives function by......

a) binding to DNA.

b) binding the ribosome

c) binding to DNA replication proteins.

d) binding PBP proteins. *

8. What is optical density?

a) A measure of the color of a solution.

b) A measure of the turbidity or cloudiness of a solution. *

c) A measure of the salt concentration in a solution.

d) A measure of the amount of protein in a solution.

9. Adding bacteriostatic antimicrobial/antibiotic to a bacterial cell culture, this would happen:

a) Optical density would go up

b) Optical density would go down

c) Optical density would stay the same *

d) None of the above

10. An enzyme does the following

a) catalyzes the production and breakdown of compounds.

b) Is involved in the replication of DNA.

c) is involved in the storage and release of energy.

d) All of the above. *

11. The active site of an enzyme is where

a) the enzyme binds catalyzes a chemical reaction.

b) the enzyme interacts with its substrate.

c) from where the enzyme releases its product.

d) All of the above. *

12. If an antimicrobial/antibiotic enters the active site of an enzyme it will inhibit activity

a) by causing the protein to aggregate

b) by causing the protein to unfold (denature)

c) by stopping the release of the product.

d) by displacing the substrate. *

13. An drug that enters the active site of an enzyme is known as :

a) An uncompetitive inhibitor

b) A non-competitive inhibitor

c) A competitive inhibitor *

d) None of the above.

14. During non-competitive inhibition.....

a) The drug causes the enzyme to denature.

b) The drug enters active site, blocking entry of substrate.

c) The drug causes a pH change in the protein.

d) The drug causes a conformational change in the enzyme. *

15. Docking software is used...

a) to model a complex of a small ligand to a protein *

b) By driver-less cars to parallel park.

c) To model the degradation of proteins.

d) To monitor red blood cell absorption of oxygen.

**Appendix 2: Student guide handout for 1) Culturing activity and 2) molecular docking activity**

**Activity 1: Culturing activity**

Identifying the mechanism of action of the antibiotics Penicillin and CKI by testing for lytic activity in cell cultures of *S. pneumoniae.*

Ideas you will become familiar with:

1. Lysis and autolysis
2. Antibiotic
3. Enzymatic activity
4. Bacteriostatic and bacteriocidal
5. Inhibitor mechanism of action

**Methods and Materials**

**Testing for lytic activity**

You will be adding CKI and penicillin, separately and in combination to cultures of *S. pneumoniae* grown to an O.D.600 of 0.5. You will then incubate the cultures in a water bath set to 37 degrees. You will then monitor the optical density of the cultures by taking absorbance readings at 600 nm for every 10 minutes placing the culture back in the water bath after every reading.

*Materials:*

1. 3x 7 mL plastic tubes containing cultures of bacteria grown to O.D600 of 0.5 (Question, why are the cells grown to an O.D. of 0.5?)
2. 1x tube (labelled BHI) containing pure culture media.
3. Water bath set at 37 degrees Celcius
4. 1 mg/mL solution of penicillin
5. 30 mM solution of CKI
6. labelling tape
7. marker
8. pipetters
9. 200 uL pipette tips (sterilized)
10. Timer
11. Spectrophotometry set to 600 nm

*Method:*

1. Add tape to the top of 3 tubes containing *S. pneumonia* culture and label using a marker (CKI, PEN, and CKI/PEN).
2. Add 5 uL of penicillin solution to the tubes marked PEN and CKI/PEN
3. Add 5 uL of CKI solution to the tubes marked CKI and CKI/PEN.
4. MARK THE TUBES WITH YOUR INITIALS
5. Blank the spectrophotometer using tube containing pure culture media (BHI)
6. Set timer to 10 minutes
7. After ten minutes measure the O.D. in the spectrophotometer.
8. Take measurements every ten minutes for an hour.
9. Record your O.D.s for every solution.

**Observations and results**

1.
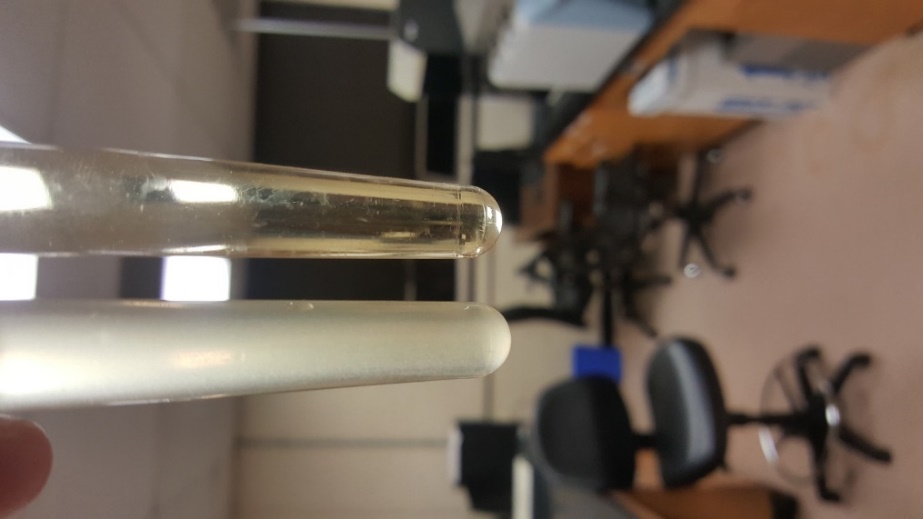
Record your O.D. results

| Time/condition | Control | PEN | CKI | PEN/CKI |
| --- | --- | --- | --- | --- |
| 0 |  |  |  |  |
| 10 |  |  |  |  |
| 20 |  |  |  |  |
| 30 |  |  |  |  |
| 40 |  |  |  |  |
| 50 |  |  |  |  |
| 60 |  |  |  |  |

1. Do you notice any trends in the data?
2. Plot the data (dot-plot) in an excel sheet (homework)

**Discussion**

**Questions to answer in your lab report:**

Activity 2: Modelling the mechanism of inhibition of by performing a docking study of the ability of CKI to enter the active site of the choline kinase of S. pneumoniae.

Ideas you will become familiar with:

1. Docking software
2. Active site
3. Energetically favorable structure
4. Competitive inhibition
5. Substrate

**
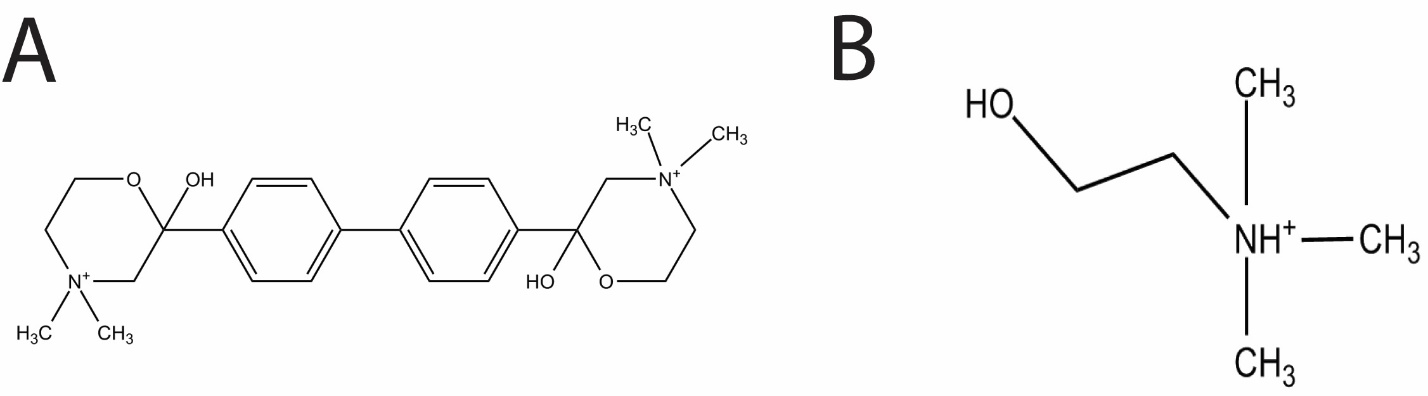
**

**Figure 1, Structures of (A) CKI and (B) choline. By definition, choline naturally enters into the active site of choline kinase (CK). Are there any similarities in the molecular structures in A and B that lead you to believe that CKI could also fit into the binding site? Differences that make you think the opposite?**

Materials:

1. PC with the visualization software Pymol, (any) web browser, and notepad installed

Methods:

The goal of this activity is to model the complex of CKI with choline kinase, using the **active site** of CK as a possible binding site. We are going to use **docking software** to determine if CKI can FIT into the active site (see Figure 2) If CKI can enter the binding site in an energetically favorable configuration, this means that it COULD function as a competitive inhibitor.


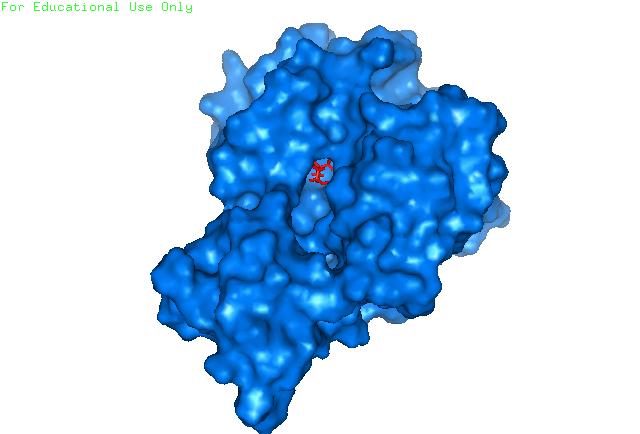

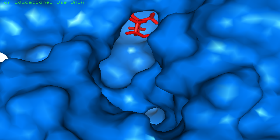

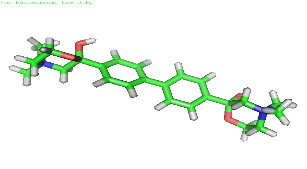


CKI

**Figure 2: The active site is where the natural subtrates of en enzyme interact with the enzyme and are converted to their products. In the case of choline kinase (CK), this means that choline enters the active site and is converted into phosphocholine. Can you tell where the active site is? Could CKI fit into the active site?**

This activity consists of six parts 1) inspection of the structure of choline kinase with choline; 2) definition of the choline kinase active site; 3) inspection of CKI; 4) docking of CKI to the active site of choline kinase; and 5) inspection of structure of choline kinase in complex with CKI; 6) comparison with the structure of choline kinase in complex its natural substrate choline **Part 1: Inspection of CK complex with choline**

1. Open Pymol
2. Load CKS_choline
3. Command: hide all
4. Command: bg white
5. Command: show surface
6. Command: show sticks, resid 301 ---This is to visualize the choline molecule
7. Inspect the space that contains the choline molecule (resid 301), what do you see?

**Part 2: Defining the active site**

1. Command: select br. all within 4 of resi 301 -----this selects residues with four Å of choline
2. Command: create activesite, sele ---- this defines a separate object of those resides
3. For the activesite, toggle the “L” and choose label residues
4. Command: hide surface ---- to see the labels of the residues
5. Write the numbers of the amino acids into a text file in a column. Each number should be followed by a space and then the letter “A”. Example: 1 A

2 A

Name text file **activesite**

1. Command: show sticks, activesite ---- inspect the defined activesite.

Is there a pattern in the residues that surround choline?

1. Hide all

**Part 3: Inspection of CKI**

Load CKI file

1. Command: show sticks, CKI
2. Inspect the CKI molecule.

**Part4: Docking the CKI into the CK protein that lacks choline**

1. Go to the Patchdoc website <https://bioinfo3d.cs.tau.ac.il/PatchDock/>
2. For receptor molecule, upload the file “CKS_apo” as the receptor molecule.
3. For ligand molecule upload file CKI
4. Click on “advanced options”
5. For receptor site, upload file **activesite.txt.**
6. Put your email address in the email address field.
7. Click on Submit
8. Check your email
9. Click on link in email
10. **Go to REFINE best solutions with** [**FireDock**](http://bioinfo3d.cs.tau.ac.il/FireDock/index.html)
11. Leave the default 10 solutions and click Go
12. Go back to your email and click on FireDock link
13. Click on “download best structures”
14. Open Zip file
15. Copy sol_1.pdb to the folder “autolysis files”

**Part5: Inspection of docked sequence**

1. Open sol_1.pdb with PyMOL
2. Command: hide all
3. Command: show surface, sol_1 --- to visualize protein surface of docked structure.
4. Click on menu---sequence---show sequence
5. Click on HC6, choose option “show sticks”.--- this is to see the CKI in the docked structure
6. Click on HC6, choose option “color blue”---- to better see the CKI molecule
7. Inspect the structure, is the ligand inside the active site?
8. Command: align sol_1, CK_choline---This commandoverlays the modeled structure of choline kinase bound to CKI (sol_1) with real structure of choline kinase bound to choline (CK_choline).
9. Command: hide everything, CK_choline
10. Click on menu---sequence---show sequence
11. Click on CHT in the sequence, from the menu, click on “show sticks”
12. Inspect the alignment, does the CKI molecule overlap with the choline molecule? Do you think the CKI could displace the choline molecule? Why?
13. File --Save image—PNG into folder Autolysis files.
14. Save the image for your lab report. Put it in your lab report. I want to see it.

**Appendix 3: Lab Report Rubric**

**Lab Report, 3 pages**

**Hypothesis: Culturing Activity**

**What are antibiotics/antimicrobials?**

Why are they important for health?

Why are they important for food safety?

What are two ways of classifying antimicrobials?

What question are we trying to answer with this culturing activity?

**Based on what we know what is the likely answer to the question [hypothesis]? What will be the effect of CKI? What will be the effect of penicillin. What background knowledge leads you to believe this.**

**What are we going to do to answer that question?**

**Hypothesis: Molecular Docking Activity**

1. **What is the question that we are trying to answer [hypothesis]?**
2. **Looking at the structure of choline and CKI (see Figure 1), do you think that CKI could enter the active site of choline kinase (CK)?**
3. **How do we define an active site?**
4. **How do we decide where the active site is?**
5. **If CKI CAN enter the active site, does this mean that it DOES enter the active site?**
6. **If an inhibitor DOES enter the active site, what kind of inhibitor is it?**
7. **If it doesn’t enter the active site what kind of inhibitor is it?**
8. **What are we going to do to answer these questions?**
9. **What is an energetically favorable structure and how is that calculated?**
10. **Based on what we know what is the likely answer to the question [hypothesis]?**

**Materials and Methods.**

What are we trying to measure in activity 1?

What are we trying to show in activity 2:?

Explain the purpose of each material and what each method measures (see student guide and laboratory slides and videos). Do not skip any material, or any method. FOR EXAMPLE, what is the O.D. 600 measuring? What are we adding the cultures and why? What about the software we used?

**Results**

**Figures:** show results with tables in the case of the autolysis activity, and with the final structure in the case of the docking activity.

Explain the results, what do you observe?

Is penicillin bacteriostatic or bacteriocidal? How about CKI? What happens when you add both antibiotics to the bacterial culture?

What does the O.D. tell you about autolysis, how can it be used to measure autolysis?

Can CKI enter the active site? How do you know? What kind of inhibitor could it be (competitive, non-competitive?

**Discussion**

Did you get the expected result with penicillin in the autolysis assay? Why? How does penicillin work and how is that reflected in the result you observed?

Did everyone who did the experiment get the same result? What does that tell you?

What enzyme does CKI inhibit? What pathway is that enzyme involved in?

What do the results in the autolysis assay with CKI tell you about how CKI functions?

What do the results with the CKI/penicillin combination tell you about how CKI functions

What experiments would you do to further confirm what kind of inhibitor CKI is (bacteriostatic vs bactericidal and competitive vs non-competitive).

How does this all relate to food safety?

**Appendix 4: Important Links**

**Kahoot.it exam:** https://create.kahoot.it/v2/share/cf550be2-8791-4917-be3e-f0820e3b45f3

**Docking site: Patchdoc website:** <https://bioinfo3d.cs.tau.ac.il/PatchDock/>

**Pymol download:** https://pymol.org/installers/PyMOL-2.4.0_0-Win64-portable-py27.zip

List of links leading to YouTube Videos reviewing some lab safety rules and steps for Activity 1: Lytic activity.

Safety Review

Safety Review Part 1 (cell plating): <https://www.youtube.com/watch?v=a6bvs3h5V9E>

Safety Review Part 2 (cell plating) : <https://www.youtube.com/watch?v=MIvJz1o2L6o>

Culturing activity Activity

Part 1: https://www.youtube.com/watch?v=nIxzWZVKVtI

Part 2: https://www.youtube.com/watch?v=1B4VoMvc8Bs

**Appendix 5: Materials and solution recipes**

**Materials necessary**

Brain Heart Infusion Powder (Accumedia catlog #7116B)

Hemocholinium-3 (Sigma catalog #A1108)

Penicillin-G (Fisher Biotech catalog # BP914-100)

Streptococcus Pneumoniae R6 (ATCC BAA-255)

8-10 mL Culture tubes

Catalase (Sigma catalog # SRE0041)

**Solutions and Media**

BHI Infusion Media

**Per Liter dH_2_0:**

37 g BHI powder

Fill a 2 L beaker with 1L dH_2_0. Place on a magnetic stirrer, and add stirring magnet. Add the BHI powder. Let the mixture stir until no reagent is clumped at the bottom and the solution is clear. (30 minutes).

Split into 1 L bottles, place bottles in an autoclave and autoclave according to instructions (60 minutes). Remove from autoclave and set to down to cool (about 45 minutes).

10 mM CKI Solution

Add 41.45 mg of Hemocholinium-3 into 10 mL water. Mix by agitation. Filter using a 0.22 μM syringe filter.

1 mg/mL Penicillin-G solution

Add 10 mg of penicillin in 10 mL of water. Mix by agitation. Filter using a 0.22 μM syringe filter.

Streptococcus Pneumoniae Cultures

Five μL of a glycerol stock of the *S. pneumoniae* R6 strain was used to inoculate 5 mL of Brain Heart Infusion broth supplemented with 5 units/mL catalase. This starter culture was incubated at 37 °C in a water bath until an optical density at 610 nm (OD_610)_ of 0.5 is reached, as measured by a spectrophotometer. Forty μL of this starter culture is used to inoculate 40 mL of BHI-CAT media with then be incubated at 37 °C in a water bath until an OD_610_ of 0.5 is reached as measured by a spectrometry. Cultures should be

**Equipment**

Biosafety hood

Autoclave

Visible range Spectrophotometer

Water bath

Appendix 6: Information on the Docking Activity

Docking software fits the molecule into the active site by trying many different molecular orientations, known as poses, to find out which orientation is the most energetically favorable. This is analogous to checking how well two puzzle pieces fit together by trying to fit the pieces in every possible orientation to see which works best. The best puzzle piece fit corresponds to the most energetically favorable fit between two molecules. Docking software calculates the energy of each fit by summing the energies of each hydrogen bond, as well electrostatic and hydrophobic interactions that form in the interface between the two molecules. An energetically favorable fit in the active site suggests that it is possible for the enzyme inhibitor to enter the active site. In addition, the model of inhibitor/enzyme complex can be inspected visually to see if the substrate and the inhibitor share the same space, which would be consistent with a competitive interaction.

This activity consisted of six parts: 1) inspection of the structure of choline kinase with choline; 2) definition of the choline kinase active site; 3) inspection of CKI; 4) docking of CKI to the active site of choline kinase; and 5) inspection of structure of choline kinase in complex with CKI; 6) comparison with the structure of choline kinase in complex its natural substrate choline (Appendix 2). Parts 1, 2, and 3 were performed using the visualization software Pymol (see Appendix 7 for download link. Parts 4 and 5 were carried out using the online software Patchdock and Firedock [7]. To perform the docking, a file with the relative coordinates of all the atoms of choline kinase (.pdb file) was uploaded into Patchdock (see Appendix 7 for link). Patchdock generated several possible solutions. Students were instructed to choose the most energy favorable structure found at the top of the list of solutions and submit this to Firedock for refinement. Firedock was found on the same website.

Part 5 and 6 visulalizations were carried using Pymol (Appendix 2) By overlaying the modelled structure with the structure of choline kinase bound to its natural substrate students were able to visualize if CKI shared the same space of the choline kinase substrate choline. Sharing the same space was considered support for the model that a competitive model of inhibition was possible.

**Appendix 7: Laboratory report rubric**

Total points: ______/ 20 possible points

| **Scale** | **Exceeds Expectations**  **5 points** | **On average, meets expectations**  **2-4 Points** | **Does not meet expectations**  **0-1 Points** |
| --- | --- | --- | --- |
| Quality of Writing  Points earned: | The laboratory report is written with completely correct scientific terminology, and very good grammar. | The laboratory report is written with somewhat correct scientific terminology and somewhat correct grammar | The laboratory report contains many. Grammatical errors and incorrect scientific language. |
| Presentation of data.  Points earned: | Results were clearly summarized in attractive and easy to understand figures and tables. Text explaining the data concise and extremely well written. | Results were somewhat well presented. Figures and tables were used, but not entirely clear. | Results were poorly presented, figures and tables were absent or inappropriate. |
| Comprehensiveness | All the questions on the laboratory outline fully and correctly answered and justified with scientific background and knowledge. | Some questions were fully and correctly answered and justified with scientific background and knowledge. | Few or no questions were correctly answered with explanatory scientific background and knowledge. |
| Discussion/Analysis  Points earned: | Analysis of results was very logical, well-argued, and related to the original hypothesis. | Analysis of results was somewhat logical and well-argued, and somewhat related to the original hypothesis. | Analysis of results was illogical poorly-argued, out of context with the original hypothesis. |

**Appendix 8: BSL-2 norms**

1. Smoking is not permitted in the building.
2. Wash your hands when entering the laboratory.
3. Disinfect your work area before and after working using an appropriate disinfectant (e.g. 70% ethanol).
4. NO food or beverages can be consumed in the Microbiology laboratory. Food and beverages for study cannot leave the laboratory.
5. Do not put hands near mouth or face when working hazardous materials. Do not apply makeup or contact lenses in the laboratory. This should be done in the restroom or outside the laboratory after washing hands.
6. All visitors to the Microbiology laboratory must see Dr. Ibrahim or Dr. Zimmerman and be accompanied at all times by someone working in the Microbiology laboratory.
7. All accidents occurring in the Microbiology laboratory should be reported to the immediate supervisor. A form should be filled out by the immediate supervisor for all accident resulting in physical harm or exposure to potentially hazardous biological or chemicals agent.
8. Microbial cultures, oligonucleotides, and toxins cannot be removed from the Microbiology laboratory.
9. Wear protective equipment when handling hazardous materials.
10. Use gloves when handling pathogens or toxins.
11. Always wash hands after removing gloves.
12. Wear eye protection while handling hazardous materials, including acids, pathogens and toxins.
13. Place all biohazard waste (including contaminated Petri dishes, used gloves, and used disposable diagnostic kits, animal carcasses) in biohazard bags.
14. Place biohazard bags in the appropriate rack for use in the laboratory.
15. Never place a un-autoclaved biohazardous waste in the trash can.
16. Once full, biohazard bag must be securely tied and taped with autoclave tape.
17. All biohazard waste in bound biohazard bags must be heated in an autoclave (set 4) at a minimum of 121ºC for at least 30 minutes at 50-80 psi.
18. Label autoclaved biohazard bags with date and name of the person handled.
19. Autoclaved bio safety bag must be dispose to the regular dumpster with wastes.
20. Do not take pathogens, toxins or inoculated specimens outside of the Microbiology laboratory, unless accompanied by appropriate signed personnel.
21. Open-toe shoes and shorts are not permitted.
22. Wear a lab coat (regular or disposable) when handling any hazardous material. Arms, legs feet, hands and eyes should be covered.
23. Aprons or lab coat should be worn when cleaning glassware and preparing culture media.
24. Do not wear lab coats or aprons, which were worn in the Microbiology laboratory, outside the Microbiology laboratory.
25. Autoclave disposable aprons/coats before discarding; autoclave reusable lab coats before washing them.
26. Date received and initials should be written on containers of newly received media and chemicals.
27. Label all items in the laboratory with name and date.
28. Do not sit on benches or desks.
29. Do not leave contaminated materials sitting on lab benches and carts. Sterilize immediately and discard.
30. When working with pathogens, remove gloves and wash hands or remove gloves before opening doors or using phones, including cellular phones. Cellular phones should not be place on laboratory benches.
31. Wash your hands before leaving the Microbiology laboratory.
32. Spill on carts should be promptly disinfected.
33. Promptly clean spill on hot plate/stirrers.
34. After using equipment (balances, pH meters, shakers, vortex, etc.) thoroughly clean and return to designated area.
35. Avoids leaving clean dry glassware on carts and drying racks above the sink.
36. After glassware, chemicals, media equipment, etc. have been cleaned, return to original storage area. Avoid creating new storage areas just because there is an empty spot-on shelf.
37. Allow screw caps to dry before putting into storage. (When screw caps are stored before dry, the cap liners may mold.)
38. To help prevent dust from collecting in and on glassware, sliding doors should be kept closed.
39. To disinfect spills containing biohazards, use a 70 percent solution of ethyl or isopropyl alcohol, a 10 percent (1:10) solution of bleach water, with a contact time of 15 minutes; or autoclave.
40. For spills that occur within a bio safety cabinet (BSC), do not turn the cabinet off. Begin the clean up immediately by treating the work surface, walls and any equipment with the BSC with disinfectant and allow to stand for 20 min. Once disinfection is completed, all the gloves and cleaning materials (sponges, paper towels, etc.) should be bagged and autoclaved.
41. If spills containing microorganisms, remove contaminated clothing and wash hands and face with soap and water. Clean the spill in the following manner:

. Pour disinfectant around the perimeter of the spill- don’t pour it directly on the spill

. Cover the spill with paper towels or similar material that has been soaked in disinfectant such as a 10 percent (1:10) solution of bleach water.

. Allow to stand for 20 minutes before wiping up.

. Place all materials used for clean-up in an autoclave bag and autoclave.

. Wash hands thoroughly.

. Report spill to the researcher in charge of the lab

40. Decontaminate culture flasks and/or plates with a 1:10 bleach/water solution or Ethanol (70%) (Allowing at least 15 minute contact time) or autoclave at 121 C for 60 minutes at 50-80 psi and return to where they are stored in the glassware cabinets.  Decontaminated plates and other decontaminated disposables and the autoclavable bags that they were autoclaved in are then placed in a black trash bag and then placed in the trash can dedicated for the purpose. This trash can be picked up and emptied by the janitorial services”.

Appendix 8: Student Performance Rubric

Total points: ______/ 20 possible points

| **Scale** | **Exceeds Expectations**  **5 points** | **On average, meets expectations**  **2-4 Points** | **Does not meet expectations**  **0-1 Points** |
| --- | --- | --- | --- |
| Engagement/Enthusiasm  Points earned: | Student adds to the class discussion and answers questions with well thought out responses. Student is enthusiastic during the laboratory activity and asks questions. | Student sometimes adds to the discussion. Student is somewhat at ease with the laboratory activity. | Student never adds to the discussion makes, or answers questions in class or laboratory. Student is uncomfortable with the activity. |
| Following instruction  Points earned: | Student always follows instructions pertaining to exercise | Student generally follows instructions, occasionally needed reminding. | Student failed to follow instruction. |
| Attendance  Points earned: | Regular and punctual attendance. | Several absences or instances of being late. | Student has regular absences and is late regularly. |
| Completion | Successful completion of the exercise, including all homework. Diligent and complete documentation of results. | Partial completion of the tasks. Moderately good documentation of results. | Tasks were left incomplete. Little or no documentation of results. |

**Appendix 10: Assessment of student outcomes**

A common way of assessing student learning is using pre- and post- test. On the multiple choice pre- and post- exams on Kahoot, the students scored 7.6 +/- 2.7 out of 15 (n=12) on the pre-laboratory exam and 12.7 +/- 1.7 out of 15 (n=12) on the post-laboratory exam, a statistically significant difference (p < 0.05). These results indicated that while students in the course had some prior knowledge of the subjects being tested, the laboratory experience reinforced that knowledge. This increase in exam scores provided evidence that students learned to the concepts behind LO1 and LO2(see Table 1). Its important to note that the enthusiasm of the students was particulary high both during the pre and post exams due to the game show nature of the Kahoot.it tool and the use of student cell phones.

The primary assessment of student outcomes was through the laboratory reports. On average students scored highly on all scales of the laboratory report assessments, with the exception of a somewhat limited average score for writing81 +/- 5.5, indicating a need for improvement in writing skills (Table 2). Meanwhile, on their classroom assessments, students scored highly on Presentation of Data (81 +/- 9.3), Comprehensiveness (92.5 +/- 8.5), Discussion/Analysis 92.0 +/- 8.5. These results indicated that the students had achieved a concepts and material imparted to them (LO2,-LO4, achieved a measure of competence in the laboratory and computational methods (LO5-LO6). The results also indicated that they understood how to analyze the results and draw conclusions from them, which was the most critical learning objective (LO7). In sum, these results showed that the students had generally understood the antibiotic/antimicrobial material that was presented to the students in the form of video, lecture, and hands-on work.

| **Learning Objective (LO)** | **Assessment method** |
| --- | --- |
| 1) Explain bacteriostatic/bacteriolytic mechanisms of action.  2) Explain competitive/non-competitive mechanisms of action.  3) Explain how turbidity changes can determine mechanism of action  4) Explain the relationship between the binding site of an antimicrobial and competitive and non-competitive mechanisms of action.  5) Apply docking software to assess binding site of an antimicrobial.  6) Apply spectrophotometric techniques in the laboratory.  7) Analyze laboratory results and draw conclusions. | Pre/Post exam, Laboratory  Pre/Post exam, Laboratory report  Laboratory report  Laboratory report.  Laboratory report  Laboratory report  Laboratory report |

**Table 1: Learning objectives of the activity and methods used to assess outcomes.**

| Student Laboratory Report Scale | **Average Percentages** | **Student Performance Scale** | **Average Percentages** |
| --- | --- | --- | --- |
| Writing | 81 +/- 5.5 | Engagement/Enthusiasm | 95 +/-6.3 |
| Presentation of Data | 89 +/- 9.9 | Following Instruction | 90 +/- 5.7 |
| Comprehensiveness | 90 +/- 7.7 | Attendance | 100 +/- 0.0 |
| Discussion/Analysis | 92 +/- 8.5 | Completion | 100 +/- 0.0 |

**Table 2: Student Laboratory Report and Student Classroom Performance Assessments (n=12). Average percentages of students each of the subscales is shown (+/- 1) standard deviation.**

Statistical Analysis

The paired t-test was used to calculate the difference within each before-and-after pair of measurements (pre- and post-laboratory exam data).  Differences between the means of data from two related samples

were considered significantly different when p < 0.05.
